# Optimal Timing for Elective Cesarean Deliveries: Insights from a Comparative Study at 38- or 39-Weeks Gestation: Elective Cesarean Delivery at 38 vs 39 Weeks

**DOI:** 10.31661/gmj.v14i.3839

**Published:** 2025-11-05

**Authors:** Shaghayegh Moradi Alamdarloo, Fariba Hoseini, Atefe Hashemi, Homeira Vafaei, Maryam Zolghadr, Hamide Barzegar

**Affiliations:** ^1^ Maternal-Fetal Medicine Research Center, Shiraz University of Medical Sciences, Shiraz, Iran; ^2^ Department of Obstetrics and Gynecology, Shiraz University of Medical Sciences, Shiraz, Iran; ^3^ Neonatal Research Center, Shiraz University of Medical Sciences, Shiraz, Iran

**Keywords:** Cesarean Section, Pregnancy, Elective Surgical Procedures, Pregnancy Outcome, Maternal Health

## Abstract

**Background:**

Background: The optimal timing for elective cesarean deliveries remains a subject of debate, with concerns regarding consequences for mother and child at different gestational ages.

**Materials and Methods:**

Materials and Methods: This prospective cohort study was done in 2022 at hospitals associated with Shiraz University of Medical Sciences. Singleton pregnant women whose pregnancies were terminated at 38(38+0 to 38+6) or 39 (39+0 to 39+6) weeks gestation were enrolled. Data on demographic characteristics, maternal and neonatal outcomes, and complications were collected from hospital records. The maternal and neonatal pregnancy outcomes were compared between the two groups using SPSS version 21. T-test, Mann–Whitney U test, Chi-square test, and Fisher’s exact test were performed.

**Results:**

Results: Among 1812 cesarean deliveries, 1274 (70.3%) were performed at 38 weeks gestational age and 538 (29.7%) at 39 weeks gestational age. Maternal complications, including infection at the surgical site (1.6% vs 0.4%, P=0.03) and uterine rupture( 0.6% vs 0, P=0.04), were more prevalent in the 38-week group. Growth indices were higher in neonates born at 39 weeks (length 49.55±2.36 vs 48.71±2.05 cm P=0.001 & weight 3.57±0.67 vs 3.37±0.59 kg P=0.001). No significant differences were observed in other maternal or neonatal complications between the two groups.

**Conclusion:**

Conclusion: Deliveries at 38 weeks were associated with a higher rate of maternal complications, possibly due to their emergent nature. However, there were no significant differences in most maternal and neonatal outcomes. Elective cesarean at 38 weeks may still be reasonable to avoid emergency operations and their risks.

## Introduction

There has been a surge in the rate of cesarean section over the years, reaching approximately 30% in high-income countries [1], a figure that exceeds the World Health Organization's recommended threshold of 10-15% for medical indications [2]. Unfortunately, various studies indicate a notably high rate of cesarean sections in Iran. Statistics reveal rates ranging from 26% to 60% in some private hospitals. Notably, published data from 1997showed a cesarean section rate of 19.5%, which surged to 42.3% by 2006 [3][4][5]. Unnecessary cesarean sections are associated with adverse effects, including heightened maternal and neonatal mortality [6][7], and impose negative economic consequences on families and society at large[8][9].

Determining the optimal timing for cesarean section presents a challenge. A retrospective study in China, a nation with a high rate of elective cesarean delivery, identified 39 weeks gestational age as the optimal time for cesarean delivery [10].

Both the National Institute for Health and Care Excellence guideline [11] and the Royal College for Obstetricians & Gynecologists guideline [12] assess the effect of early-term cesarean sections on neonatal respiratory complications, recommending against elective cesarean deliveries before 39 weeks and 0 days of gestation. Additionally, the American College of Obstetricians and Gynecologists [13], generally discourages indicated deliveries before 39 weeks and 0 days of gestation, with exceptions for specific pregnancy complications or comorbidities. Given the scarcity of high-level evidence, a systematic review and meta-analysis were conducted to determine the optimal timing of cesarean section, revealing increased rates of neonatal intensive care unit (NICU) admission and mortality for those delivered before 39 weeks gestation, though evidence regarding maternal complications was insufficient [14]. No maternal benefit has been demonstrated from delaying elective cesarean sections to 39 weeks gestation, but information in this area is very limited [14].

Therefore, it is important to carefully evaluate the outcomes of planned cesarean deliveries at different gestational ages, especially between 38 and 39 weeks. Observational studies may be affected by bias, as some cesareans are performed earlier due to medical concerns, which can lead to more complications being reported in these cases. To better understand the true risks and benefits of scheduling cesareans at 38 versus 39 weeks, we need studies that focus specifically on women who are candidates for elective cesarean delivery. In addition, since factors like ethnicity may affect the timing of labor and emergency cesarean risk, having more local data can help guide health policy decisions about the best timing for elective cesareans. This study aims to compare maternal and neonatal outcomes of elective cesarean deliveries performed at 38 and 39 weeks of gestation.

## Material and Methods

This prospective cohort study was conducted in 2022 at hospitals associated with Shiraz University of Medical Sciences. The study enrolled singleton pregnant women whose pregnancies were terminated at either 38 weeks gestational age (38+0 to 38+6) or 39 weeks gestational age (39+0 to 39+6), as determined by sonography according to crown-rump length measurement in 11 to 13 weeks gestational age. Based on local guidelines, elective cesarean sections were performed at 39 weeks gestation and those terminated at 38 weeks were emergent situations. Exclusion criteria included multiple pregnancies, maternal age less than 18 years old, language difficulties requiring translation, and medical issues arising in pregnancy such as gestational diabetes mellitus, preeclampsia, placenta previa, placenta accreta, hypertension, and raised hepatic enzyme levels.

Data collection was on the basis of hospital records and included demographic information as well as maternal and neonatal outcomes. All participating hospitals had 24-hour specialist obstetrician-gynecologists, pediatricians, and anesthesiologists. They adhered to national guidelines for cesarean sections, including the preference for local anesthesia over general anesthesia and the administration of preventive antibiotic therapy during the operation. A midwife was present in the operating room to attend to the neonate immediately after birth. If the neonate exhibited any symptoms, a pediatrician was summoned for assessment and care. Maternal complications, such as mortality, hysterectomy, thromboembolic events, and surgical complications (e.g., intestinal, bladder, or uterine injury, rupture) were recorded. Any complications necessitating surgery or antibiotic therapy within the first-month post-delivery were also documented. Anesthesia-related complications, such as hypo/hypertension, headache, or the need for general anesthesia, were likewise recorded.

Neonatal outcomes encompassed sex, growth parameters (height, weight, head circumference), Apgar scores, cord pH, NICU admission, duration of hospitalization, respiratory support requirements (intubation, continuous positive airway pressure, oxygen therapy), antibiotic therapy, hypoglycemia, hyperbilirubinemia, and asphyxia.

Data analysis was conducted using the Statistical Package for the Social Sciences (SPSS version 21, IBM Corp., Armonk, NY, USA). Descriptive statistics, including maximum, minimum, mean ± standard deviation, frequency, and percentage, were used for quantitative and categorical variables, respectively. The Shapiro-Wilk test was applied to evaluate the data's normality. Appropriate analytical tests, such as the t-test, Mann-Whitney U test, Chi-square test, and Fisher's exact test, were performed. A P-value of less than 0.05 was considered statistically significant.

Ethical approval for the study was obtained from the Shiraz University of Medical Science ethics committee (IR.SUMS.MED.REC.1402.300). Written informed consent was obtained from all participants prior to their enrollment in the study

## Result

**Table:** Table1. Gravidity and History of Previous Cesarean Sections Among Participants

** ** **Parameters**	**Numbers**	**Total (N= 1812)**	**39 weeks (N= 538)**	**38 weeks (N=1274)**	**P-value**
	1	610(33.7)	427(33.5)	183(34)	
	2	541(29.9)	379(29.7)	162(30.1)	
	3	380(21)	274(21.5)	106(19.7)	
	4	208(11.5)	150(11.8)	58(10.8)	
Pregnancy numbers	5	43(2.4)	31(2.4)	12(2.2)	0.11
	6	13(0.7)	7(0.5)	6(1.1)	
	7	9(0.5)	3(0.2)	6(1.1)	
	8	6(0.3)	2(0.2)	4(0.7)	
	9	2(0.1)	1(0.1)	1(0.2)	
	0	907(50.1)	637(50)	270(50.2)	
Previous sections	1	664(36.6)	467(36.7)	197(36.6)	0.99
	2	212(11.7)	150(11.8)	62(11.5)	
	3	29(1.6)	20(1.6)	9(1.7)	

Assessed by Chi-square test or Fisher’s exact test ^**^A P-value less than 0.05 is considered significant.

**Table:** Table2. Maternal Complications Following Cesarean Delivery at 38 and 39 Weeks of Gestation

**Complications**	**Total (N= 1812)**	**39 weeks (N= 538)**	**38 weeks (N=1274)**	**P-value**
Infection at the site of cesarian section	22(1.2%)	2(0.4%)	20(1.6%)	0.03
Bleeding at the site of cesarian section	21(1.2%)	4(0.7%)	17(1.3%)	0.34
Uterine rupture	11(0.6%)	0	11(0.6%)	0.04
Emboli	0	0	0	
Wound dehiscence	12(0.7%)	4(0.7%)	8(0.6%)	0.75
Complications of surgery	27(5.1%)	3(0.6%)	24(1.9%)	0.03
Complications of anesthesia	10(0.6%)	3(0.6%)	7(0.5%)	0.99
Admission in ICU	0	0	0	
Mortality	0	0	0	

ICU: Intensive Care Unit Assessed by Chi-square test or Fisher’s exact test ^**^A P-value less than 0.05 is considered significant.

**Table:** Table3. Neonatal Complications Associated with Cesarean Delivery at 38 and 39 Weeks of Gestation

**Neonatal outcome**	**Total (N= 1812)**	**39 weeks (N= 538)**	**38 weeks (N=1274)**	**P-value**
Hyperbilirubinemia	884(48.8%)	265(49.3%)	619(48.6%)	0.79
Hyperbilirubinemia requiring hospital admission	28(5.1%)	6(1.1%)	22(1.7%)	0.4
NICU admission	15(0.8%)	3(0.6%)	12(0.9%)	0.57
Asphyxia	1(0.1%)	0	1(0.1%)	0.99
Bradycardia	4(0.2%)	1(0.2%)	3(0.2%)	0.99
Hypothermia	12(0.7%)	7(1.3%)	5(0.4%)	0.05
Hypoglycemia	6(0.3%)	2(0.4%)	4(0.3%)	0.99

NICU: Neonatal Intensive Care Unit Assessed by Chi-square test or Fisher’s exact test ^**^A P-value less than 0.05 is considered significant.

**Table:** Table4. Apgar Scores and Neonatal Growth Parameters at Birth by Gestational Age

**Neonatal outcome**	**Total (N= 1812)**	**39 weeks (N= 538)**	**38 weeks (N=1274)**	**P-value**
1^st^ min. Apgar Score	9.9±0.043	9.91±0.039	9.9±0.044	0.56
5^th^ min. Apgar Score	9.93± 0.028	9.93±0.027	9.93±0.028	0.74
Length (cm)	49.3± 2.3	49.55±2.36	48.71±2.05	0.0001
Weight (kg)	3.51±0.65	3.57±0.67	3.37±0.59	0.0001
Head circumference (cm)	34.65±1.15	34.67±1.02	34.77±1.18	0.65

Min.: minute, cm: centimeter, kg: kilogram Assessed by independent t-test or Mann-Whitney U test ^**^A P-value less than 0.05 is considered significant.

A total of 1812 pregnant women and neonates were evaluated. The mean age of mothers was 27.85±5.52 years (P-value 0.13). Pregnancy termination was performed in 1274 cases (70.3%) at 38 weeks gestational age (38+0 to 38+6) and 538 cases (29.7%) at 39 weeks gestational age (39+0 to 39+6). The number of pregnancies and previous sections in each group are shown in Table-1. As shown, most of the women were gravida 1, experiencing their first pregnancy (610; 33.7%). As the number of pregnancies increased, the number of patients decreased accordingly. Regarding previous cesarean sections, 907 women (50.1%) had none, while 664 women (36.6%) had one previous cesarean section.

The two groups' Maternal complications were compared and shown in Table-2. Maternal complications were generally low across both groups. However, infections at the cesarean site were more common in the 38-week group compared to the 39-week group (1.6% vs. 0.4%, P=0.03). Uterine rupture occurred exclusively in the 38-week group (0.6%, P=0.04). Overall surgical complications were also higher in the 38-week group (1.9% vs. 0.6%, P=0.03). No cases of embolism, ICU admission, or mortality were reported in either group, and other complications such as bleeding, wound dehiscence, and anesthesia-related issues showed no differences.

The duration of maternal hospitalization was 1.53±0.61 days. In the 39-week group, it was 1.53±0.6 and in the 38-week group, it was 1.52±0.61 days. (P-value 0.73)

Neonatal outcomes were also evaluated, as shown in Table-3. The overall rate of hyperbilirubinemia was nearly 49% in both groups (P=0.79), with no significant difference in cases requiring hospital admission. Rates of NICU admission, asphyxia, bradycardia, and hypoglycemia were low and comparable between groups. Hypothermia showed a borderline higher incidence in the 39-week group compared to the 38-week group (1.3% vs. 0.4%, P=0.05).

Neonates were also compared for their Apgar Score of 1 and 5 minutes, and growth indices as shown in Table-4. The Apgar scores at 1 and 5 minutes were similar between groups (P=0.56 and P=0.74, respectively). However, neonates delivered at 39 weeks had greater length (49.55 ± 2.36 cm vs. 48.71 ± 2.05 cm, P=0.0001) and weight (3.57 ± 0.67 kg vs. 3.37 ± 0.59 kg, P=0.0001) compared to those delivered at 38 weeks. Head circumference did not differ significantly between groups (P=0.65).

## Discussion

This study highlights important differences in maternal and neonatal outcomes between cesarean deliveries performed at 38 versus 39 weeks of gestation. Overall, cesarean sections at 39 weeks were associated with fewer maternal complications and more favorable neonatal anthropometric measurements. While certain maternal complications, such as surgical site infection and uterine rupture, appeared more frequently at 38 weeks—potentially due to a higher rate of emergent procedures—most other maternal and neonatal adverse outcomes were comparable between the two groups. These findings support the potential benefits of scheduling elective cesarean deliveries at 39 weeks when clinically feasible, in line with recommendations aimed at optimizing both maternal and neonatal health.

Our study demonstrated a significantly higher incidence of maternal complications—particularly infection and uterine rupture—in women who underwent cesarean delivery at 38 weeks of gestation. This increase may be attributed to the higher proportion of emergency cesarean deliveries performed before 39 weeks, where clinical urgency often limits optimal preoperative preparation and elevates surgical risk. These findings are consistent with a study by Yang et al., which reported that emergency cesarean sections were associated with a greater incidence of maternal complications, including infection, fever, urinary tract infections (UTIs), wound dehiscence, disseminated intravascular coagulation (DIC), and the need for reoperation, in comparison to elective procedures. Interestingly, headaches were the only complication more frequently observed in elective cases [15].

In our study, all 11 cases of uterine rupture occurred in women who underwent cesarean delivery at 38 weeks of gestation. Uterine rupture is primarily associated with risk factors such as a previously scarred uterus—often following a prior cesarean section—and the use of prostaglandins for labor induction [16]. A multicenter study conducted across 19 academic institutions over a three-year period similarly found that uterine rupture was rare in elective cesarean deliveries. However, women undergoing cesarean delivery for medical indications faced a significantly higher risk of uterine rupture and adverse maternal and neonatal outcomes [17]. In our cohort, all uterine ruptures occurred during emergency cesarean deliveries, further supporting this association. These findings suggest that appropriately scheduled cesarean sections—preferably at or beyond 39 weeks—may reduce the likelihood of emergency procedures and the complications that may follow, including uterine rupture.

Regarding neonatal outcomes, our study found no statistically significant differences between the two groups in terms of NICU admission, asphyxia, hypothermia, hypoglycemia, bradycardia, or Apgar scores. However, neonates delivered at 39 weeks of gestation demonstrated significantly higher birth weights and lengths compared to those delivered at 38 weeks. This finding aligns with previous research; for instance, a retrospective 7-year cohort study conducted by Wilmink et al. in the Netherlands reported neonatal complication rates of 12.5% for those born at 38-39 weeks, compared to 9.5% for neonates delivered at 39 weeks or later via cesarean section [18]. Similarly, Vidic et al. concluded that cesarean delivery at 39 weeks or later was associated with fewer neonatal morbidities compared to earlier deliveries [19]. These findings underscore the potential benefits of postponing elective cesarean deliveries until at least 39 weeks to support optimal neonatal growth and reduce the risk of complications.

Some studies consistent with our findings suggest that performing elective cesarean sections at 38 weeks of gestation does not necessarily lead to an increased rate of neonatal complications. For example, Hefney et al. found no significant differences in the need for NICU admission or respiratory support between neonates delivered by cesarean section at 38 versus 39 weeks of gestation [20]. Similarly, Glavind et al. reported comparable rates of NICU admissions exceeding two days in both gestational age groups [21]. However, conflicting evidence exists; in a study by Pirjani et al., NICU admissions were more common among neonates born at 38 weeks compared to those delivered at 39 weeks [22]. These discrepancies across studies highlight the complexity of neonatal outcomes and the importance of considering additional clinical factors when determining the optimal timing of elective cesarean delivery.

A key strength of this study is its large sample size, which enabled the comprehensive analysis of various maternal and neonatal outcomes, including rare but clinically significant complications. This robust population base enhances the generalizability and statistical power of the findings. However, one limitation is the potential for data entry errors or subjective variability, particularly in variables such as the Apgar score, which may not always accurately reflect the newborn's true clinical condition.

Therefore, our study did not reveal significant differences in most maternal and neonatal outcomes between cesarean deliveries performed at 38 versus 39 weeks of gestation. These findings offer valuable insight into the implications of rising elective cesarean rates, particularly primary cesarean sections, and promote more informed decision-making regarding the optimal timing of scheduled cesarean deliveries. Notably, our data indicated that the number of emergency cesarean sections at 38 weeks was more than double that of elective cesareans at the same gestational age. Based on our results, delaying cesarean delivery until 39 weeks may not be universally necessary or advantageous. However, further prospective, randomized controlled trials are required to more precisely evaluate the risks of postponing cesarean delivery to 39 weeks compared to earlier intervention, as well as to investigate long-term neonatal outcomes and the economic implications of elective cesarean timing.

## Conflict of Interest

None.
